# Photon-counting CT-angiography in pre-TAVR aortic annulus assessment: effects of retrospective vs. prospective ECG-synchronization on prosthesis valve selection

**DOI:** 10.1007/s10554-024-03050-w

**Published:** 2024-02-15

**Authors:** Muhammad Taha Hagar, Theresa Kluemper, Manuel Hein, Constantin von Zur Muhlen, Sebastian Faby, Fabio Capilli, Christopher Schuppert, Ramona Schmitt, Philipp Ruile, Dirk Westermann, Christopher L. Schlett, Fabian Bamberg, Tobias Krauss, Martin Soschynski

**Affiliations:** 1 Department of Diagnostic and Interventional Radiology, Medical Center – University of Freiburg, Faculty of Medicine, University of Freiburg, Hugstetter Straße 55, 79106 Freiburg, Germany; 2 Department of Cardiology and Angiology, Medical Center - University of Freiburg, Faculty of Medicine, University of Freiburg, Freiburg, Germany; 3 Computed Tomography, Siemens Healthineers AG, 91301 Forchheim, Germany; 4 Department of Radiology, Neuroradiology and Nuclear Medicine, Medical Center Vest, Ruhr University Bochum, Dorstener Straße 151, 45657 Recklinghausen, Germany

**Keywords:** Computed tomography angiography, Aortic valve stenosis, Cardiac computed tomography

## Abstract

To compare the diagnostic value of ultrahigh-resolution CT-angiography (UHR-CTA) compared with high-pitch spiral CTA (HPS-CTA) using a first-generation, dual-source photon-counting CT (PCD-CT) scanner for preprocedural planning of transcatheter aortic valve replacement (TAVR). Clinically referred patients with severe aortic valve stenosis underwent both, retrospective ECG-gated cardiac UHR-CTA (collimation: 120 × 0.2 mm) and prospective ECG-triggered aortoiliac HPS-CTA (collimation: 144 × 0.4 mm, full spectral capabilities) for TAVR planning from August 2022 to March 2023. Radiation dose was extracted from the CT reports, and the effective dose was calculated. Two radiologists analyzed UHR-CTA and HPS-CTA datasets, assessing the image quality of the aortic annulus, with regard to the lumen visibility and margin delineation using a 4-point visual-grading scale (ranges: 4 = ”excellent” to 1 = ”poor”). Aortic annulus area (AAA) measurements were taken for valve prosthesis sizing, with retrospective UHR-CTA serving as reference standard. A total of 64 patients were included (mean age, 81 years ± 7 SD; 28 women) in this retrospective study. HPS-CTA showed a lower radiation dose, 4.1 mSv vs. 12.6 mSv (p < 0.001). UHR-CTA demonstrated higher image quality to HPS-CTA (median score, 4 [IQR, 3–4] vs. 3 [IQR, 2–3]; p < 0.001). Quantitative assessments of AAA from both CTA datasets were strongly positively correlated (mean 477.4 ± 91.1 mm^2^ on UHR-CTA and mean 476.5 ± 90.4 mm^2^ on HPS-CTA, Pearson r^2^ = 0.857, p < 0.001) with a mean error of 22.3 ± 24.6 mm^2^ and resulted in identical valve prosthesis sizing in the majority of patients (91%). Patients with lower image quality on HPS-CTA (score value 1 or 2, n = 28) were more likely to receive different sizing recommendations (82%). Both UHR-CTA and HPS-CTA acquisitions using photon-counting CT technology provided reliable aortic annular assessments for TAVR planning. While UHR-CTA offers superior image quality, HPS-CTA is associated with lower radiation exposure. However, severely impaired image quality on HPS-CTA may impact on prosthesis sizing, suggesting that immediate post-scan image evaluations may require complementary UHR-CTA scanning.

**Supplementary Information:**

The online version contains supplementary material available at 10.1007/s10554-024-03050-w.

## Introduction

End-stage Aortic valve stenosis is a severe and progressive disease resulting in an obstruction of left ventricular output, heart failure, and ultimately, death due to cardiovascular disease [1]. Without treatment, the mortality in symptomatic patients exceeds 50% within two years [2]. Transcatheter aortic valve replacement (TAVR) represents an established treatment alternative to surgical aortic valve repair and is strongly recommended for patients at high-surgical risk [3, 4]. There is also growing evidence of its beneficial use in patients at intermediate or low-surgical risk [5, 6]. The preprocedural planning for TAVR relies heavily on Electrocardiogram (ECG)-synchronized CT-angiography (CTA), which provides exact aortic annular sizing and the ideal access route evaluation [7], and can be used for post-procedural risk stratification [8]. Given the dynamic changes that the aortic annulus undergoes during the cardiac cycle, manufacturers base their heart valve sizing recommendations on systolic measurements, as this is when the annulus typically reaches its largest dimension [9]. Systolic visualization of the aortic root can be achieved using Dual-Source CT technology either via low-pitch retrospective spiral cardiac CTA or prospective ECG-triggering [10]. However, current TAVR-planning guidelines still favor the use of retrospective low-pitch spiral CTA [11].

Photon-counting detector CT (PCD-CT) is an emerging technology providing improved geometrical dose efficiency, superior image quality, and spatial resolution whilst maintaining high-temporal resolution [12]. The latter is particularly crucial in cardiac CT imaging [13]. This raises the question of whether the currently guideline-recommended retrospective spiral scan remains necessary for TAVR planning in light of novel dual-source PCD-CT technology. Therefore, our study aimed to compare quantitative measurements of aortic annular sizing prior to TAVR using low-pitch, retrospective UHR-CTA, and high-pitch spectral (HPS)-CTA.

## Materials and methods

### Ethics statement

This analysis is part of a broader prospective study sanctioned by the Institutional Review Board of the ***University Medical Center Freiburg*** (approval ID, 21–2469 and approval date, 09/21/2021). It focuses on exploring the functionalities and properties of novel PCD-CT across various clinical applications, adhering strictly to the principles outlined in the Declaration of Helsinki. All study subjects provided informed written consent prior to their inclusion.

### Patient sample

In this single-center, retrospective analysis of a prospective cohort, consecutive patients with a referral for CT prior to TAVR between August 2022 and March 2023 were considered for inclusion. All patients had confirmed severe aortic valve stenosis. Additional inclusion criteria consisted of a complete scanning protocol consisting of both—UHR-CTA and HPS-CTA. Patients presenting contraindications to contrast-enhanced CTA or previous aortic valve replacement were excluded from the analysis.

### CT acquisition protocol

All study subjects were scanned on a first-generation, dual-source PCD-CT scanner (NAEOTOM Alpha, software version syngo CT VA50, Siemens Healthcare—Forchheim, Germany). The scanning protocol involved:

1. A retrospective ECG-gated, low-pitch helical cardiac CTA employing the ultrahigh-resolution Quantum HD Cardiac scan mode (collimation: 120 × 0.2 mm) with ECG pulsing set at 20–80% of the R-R interval in accordance with guideline recommendations for TAVR-CT [11]. This was followed by
2. A prospective ECG-triggered, high-pitch aortoiliac CTA employing the spectral Quantumplus mode (collimation 144 × 0.4 mm) to evaluate the feasibility of an arterial access route—technical scan parameters were previously described in detail [14]. A systolic triggering of HPS-CTA at 25% of the R–R interval was selected to depict the aortic annulus in the systolic phase.

Using a dual-syringe power injector (Accutron CT-D, Medtron AG—Saarbruecken, Germany), a combined contrast protocol was administered. This consisted of 70 ml of Iopromid (Ultravist 370, 370 mg iodine/ml, Bayer Healthcare, Leverkusen Germany), followed by a chaser consisting of 40 ml isotonic saline and 30 ml Iopromid, each delivered at aat a flow rate of 5.0 ml/sec. The UHR-CCTA was initiated first using bolus tracking with a delay of 10 s after attenuation exceeded 130 Hounsfield Units (HU) in a region of interest (ROI) placed in the aortic root. The HPS-CTA commenced immediately afterward with a minimal delay of approximately 6 s to ensure consistent image quality without administering contrast twice.

### CT reconstructions

UHR-CTA multiphase data were reconstructed in steps of 50 ms of the R–R interval facilitating the identification of the optimal systolic phase for each individual**.** All images were reconstructed using a vascular convolution kernel (Bv48, Quantum Iterative Reconstruction Level 3), a 512^2^-matrix size, and a field of view restricted to the heart of 180 × 180 mm. For HPS-CTA monoenergetic 60 keV was applied. A slice thickness of 0.6 mm with an increment of 0.6 mm was employed for UHR-CTA and HPS-CTA.

### Analysis of CT data

Two radiologists with 2 years ***(T.K.)*** and 4 years ***(M.T.H.)*** of experience in cardiovascular CT imaging assessed all images independently on a dedicated workstation (syngo.via, version VB60, Siemens Healthcare—Forchheim Germany, and 3mensio, software version 10.1, Pie Medical Imaging—Maastricht, The Netherlands). They were both blinded to clinical data and each other. All patients` Volume CT Dose index (CTDIvol) and Dose Length Product (DLP) were extracted from the CT reports. To calculate the effective dose, the DLP was multiplied with a conversion factor of κ = 0.014 mSv × (mGycm)^−1^[15]. ECG-reports during CTA acquisition were transferred as DICXOM data, maximum and minimum heart rate as well as medium heart rate were noted. To calculate heart rate variability during CTA acquisition, the following equation was applied, as previously reported: Calculation previously reported [16]: maximum heart rate – minimum heart rate/((maximum heart Rate + minimum heart rate)/2).

#### Aortic annulus assessment: subjective image quality

The subjective image quality of the aortic annulus was assessed using a 4-point visual grading scale for each patient on UHR-CTA and HPS-CTA images. Five practice cases were utilized to familiarize the evaluators before the read-out, which were not included in the study population. A score of 4 denoted “*excellent*” image quality, representing a clear and artifact-free depiction of the aortic annulus with perfect lumen and margin visibility. Image quality was deemed “*good*” (score of 3) for images with minor artifacts. A score of 2 indicated “*fair*” image quality due to detectable artifacts and a score of 1 indicated “*poor*” image quality due to the high presence of artifact interference or blurred depiction of the lumen or margin. Examples are given in Figure S1.

#### Aortic annulus assessment: objective image quality

On both UHR-CTA and HPS-CTA, at the level of the aortic root, a ROI with 15 mm in diameter was placed, and the mean CT attenuation in Hounsfield Units (HU) was registered to account for signal, as well as the standard deviation (SD) of attenuation was noted to quantify the level of noise. An ROI of similar size was placed in the paraaortic mediastinum, and the mean attenuation was registered to account for background. Signal-to-noise ratio (SNR) and contrast-to-noise ratio (CNR) were calculated using the following equations:

$$SNR=\frac{(Aortic \,Root) \,mean \,HU}{(Aortic\, Root) \,SD \,HU}$$

$$CNR=\frac{\left(Aortic \,Root\right)\,mean \,HU-(Paraaortic \,Mediastinum) \,mean \,HU}{(Aortic \,Root) \,SD \,HU}$$

#### Quantitative measurements of the aortic annulus area and the aortic annulus perimeter

The aortic annulus was characterized using a virtual semi-oval ring intersecting the basal hinge points of the three aortic valve cusps [17]. This plane was established through multiplanar reformation, adjusted to a double-oblique transverse view to intersect the cusps' most inferior attachments of the native aortic valve. Following the accurate positioning of the plane, the aortic annulus area (AAA) and aortic annulus perimeter (AAP) were determined using planimetry, and effective area- as well as perimeter-derived diameters were calculated. The systolic phase of the maximum aortic annular dimension was used to perform quantitative AAA and AAP measurements on UHR-CTA.

### Hypothetical aortic valve prosthesis selection

Based on the cross-sectional quantitative measurements of both CTA datasets, a hypothetical prosthesis valve selection was performed. Following the manufacturer’s sizing recommendations, prosthesis sizing for a balloon-expandable Edwards SAPIEN 3 heart valve (Edwards Lifesciences, Irvine, CA, USA) involved cut-off values based on the AAA. For the self-expandable Evolut R heart valve (Medtronic MN, USA), cut-off values were based on the AAP [18].

### Statistics

All statistical analyses were performed using IBM SPSS Statistics for Macintosh (version 28.0, Armonk, NY, United States). Q–Q plot and one-sample Shapiro–Wilk test were used to check for the assumption of normal distribution. Depending on their normality, variables were expressed as mean ± SD, or median and interquartile range. Variables were compared with a two-tailed t-test when normally distributed and with Wilcoxon signed-rank test when non-normally distributed. To assess which parameters would have an influence on the aortic annulus image quality, we fitted linear regression models, which included the subjective image quality as the outcome of interest and body-mass-index, CTDIvol, mean heart rate, heart rate variability, average HU attenuation, and CNR as covariates. To assess which parameters have an influence on mean annular area difference measured in both scanning techniques (UHR-CTA and HPS-CTA), a multiple linear regression model involving the same covariates was performed. Pearson’s r was used for the correlation between AAA and AAP measurements of both CTA datasets. After stratification for image quality, Bland–Altman-analysis was performed to analyze the agreement of quantitative aortic annular measurements within both datasets. Agreement between both readers for quantitative AAA and AAP measurements was determined using intraclass correlation coefficient analysis (model: two-way, type: agreement, unit: of analysis: single) and interpreted according to Koo et al. [19]. A two-tailed *P* value of  < 0.05 was considered to infer statistical significance.

## Results

### Patient sample

**Fig. 1:**
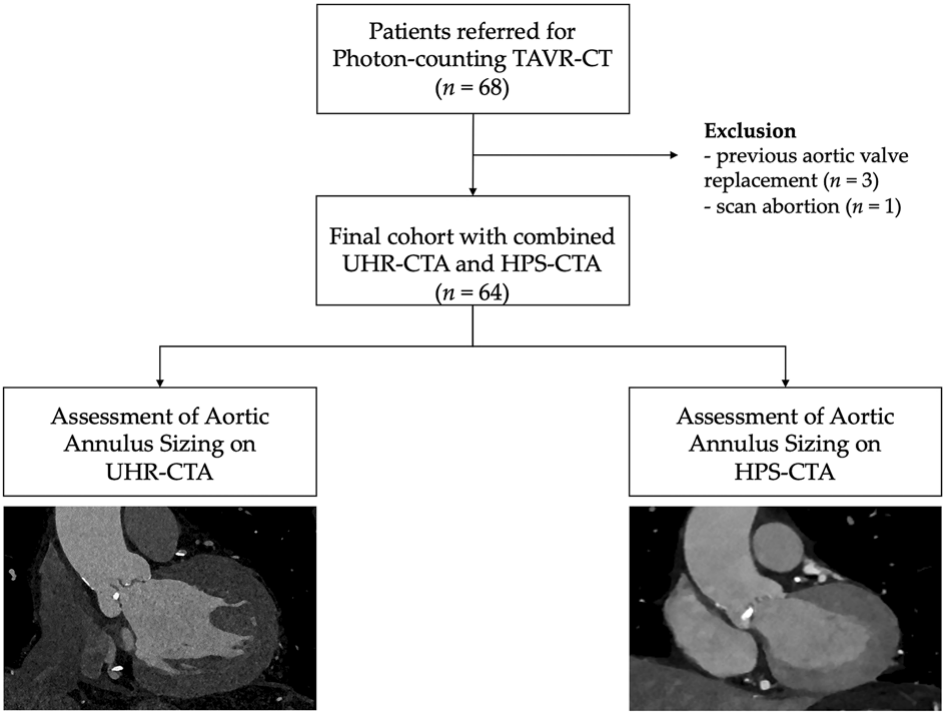
Represents the flowchart of patient inclusion and exclusion. The aortic annular area and aortic annular perimeter were measured on ultrahigh-resolution CT-angiography and on high-pitch spectral CT-angiography images. *UHR-CTA,* ultrahigh-resolution CT-angiography; *HPS-CTA*, high-pitch spectral aortoiliac CT-angiography

64 patients (mean age, 81.4 years ± 6.9 SD; 28 women) who underwent PCD-CT for TAVR planning were included. Patient characteristics are provided in Table 1. Four patients were excluded from the analysis. Information on the workflow and patient inclusion and exclusion is given in Fig. 1.

**Table 1:** Patient characteristics

Characteristics	Values
Number of patients	64 (100%)
Gender	
Male	36 (56%)
Female	28 (44%)
Age	81.4 ± 6.9
Height (cm)	168.4 ± 8.5
Weight (kg)	76.1 ± 13.8
Sinus Rhythm	47 (71%)
Heart Rate (bpm) during CTA	73 ± 13 (range 36–136)
Heart Rate Variabilty during CTA	015 [0.04–0.38]
Body-Mass-Index (kg/m^2^)	26.9 ± 4.6
Cardiovascular Risk Factors and Assessment	
Coronary Artery Disease	22 (34%)
Arterial Hypertension	53 (83%)
Diabetes Mellitus	14 (22%)
Hyperlipidemia	41 (64%)
Smoking	18 (28%)
History of Stenting	15 (23%)
Chronic Kidney Disease (eGFR ≤ 45 ml/min*1.72m^2^)	18 (28%)

Data are presented as numbers and frequencies in parenthesis, mean ± standard deviation

*eGFR* estimated Glomerular Filtration Rate, *LV-EF* left ventricular ejection fraction

^*^Value are represented in median and interquartile range in square brackets. reported: maximum heart rate–minimum heart rate/((maximum heart Rate + minimum heart rate)/2)

### Evaluation of subjective and objective image quality

**Fig. 2:**
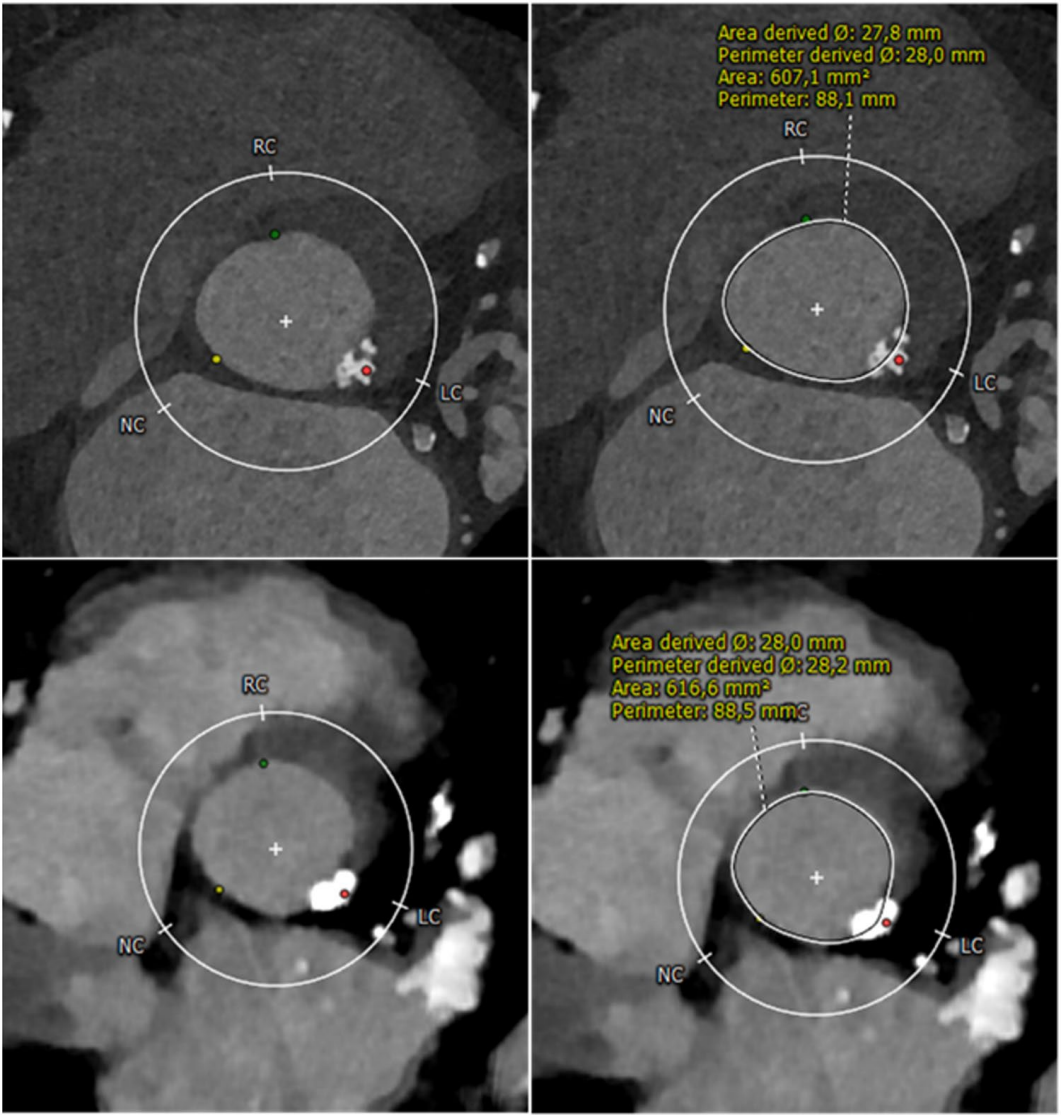
Photon-Counting detector CT angiography (CTA) of an 86-year-old male with severe aortic valve stenosis, conducted as a planning for transcatheter aortic valve replacement (TAVR). The aortic annulus was assessed with ECG-gated retrospective Ultrahigh-Resolution (UHR)-CTA (**A**, **B**) and ECG-triggered prospective high-pitch spectral aortoiliac (HPS)-CTA (**C**, **D**). Note the superior image quality of UHR-CTA and detailed visualization of the calcified plaque at the aortic annulus. Regardless, pre-TAVR CT measurements were consistent across both methods, with area-derived and perimeter-derived diameters resulting in the same hypothetical prosthesis choice. Notably, the radiation dose was higher for UHR-CTA (651 mGy*cm) than HP-CTA (297 mGy*cm). *Note:* In panel C and D of HPS-CTA, the area of increased attenuation within the left atrial appendage (arrowhead) represents increased attenuation due to accumulated contrast media

The aortic annulus on UHR-CTA demonstrated a higher subjective image quality (4 [3, 4] vs. 3 [2, 3], p < 0.001). In UHR-CTA, excellent image quality (score value of 4) was achieved most frequently – 44 of 64 subjects (68.8%), while in HPS-CTA, good image quality (score value of 3) was observed in27 of 64 (42.2%) cases. Poor image quality (score value of 1) was registered in two of 64 (3.1%) subjects scanned with UHR-CTA, while for HPS-CTA, poor image quality was noted in 11 of 64 (17.2%) individuals. In multiple linear regression analysis with subjective image quality as the outcome of interest, intraluminal attenuation was the only significant predictor (p = 0.007), as shown in Supplementary Table S1. Notably, HPS-CTA resulted in a reduced image noise (22.0 ± 7.1 vs. 31.4 ± 8.5, p < 0.001), which was reflected in objective image quality parameters, CNR (16.2 ± 5.9 vs. 14.7 ± 4.4, p < 0.001). Additionally, HPS-CTA was advantageous in terms of radiation exposure, with an Effective Dose of 4.1 mSv vs. 12.6 mSv (p < 0.001 respectively). Detailed metrics of subjective and objective image quality and radiation dose are provided in Table 2. An imaging example is given in Fig. 2.

**Table 2:** Image quality and radiation dose parameters

	Retrospective UHR cardiac CTA	Prospective High-Pitch aortoiliac CTA	p–value
Image Quality Score	4 [3, 4]	3 [2, 3]	< 0.001
Aortic Root attenuation	435 ± 84	325 ± 85	< 0.001
Image noise	31.4 ± 8.5	22.0 ± 7.1	< 0.001
Signal-to-noise ratio	14.7 ± 4.4	16.2 ± 5.9	< 0.001
Contrast-to-noise ratio	16.7 ± 4.9	19.0 ± 6.5	< 0.001
CTDIvol (mGy)	65.9 [54.6–80.1]	4.3 [3.8–5.4]	< 0.001
DLP (mGy*cm)	902 [736–1071]	292 [262–365]	< 0.001
Effective Dose (mSv)	12.6 [10.3–15.0]	4.1 [3.7–5.1]	< 0.001

Data are presented as mean ± standard deviation or median and interquartile range in square brackets. *CTDIvol* CT Dose Index volume, *DLP* Dose-Length-Product

### Quantitative measurements of the aortic annular area and the aortic annular perimeter

**Fig. 3:**
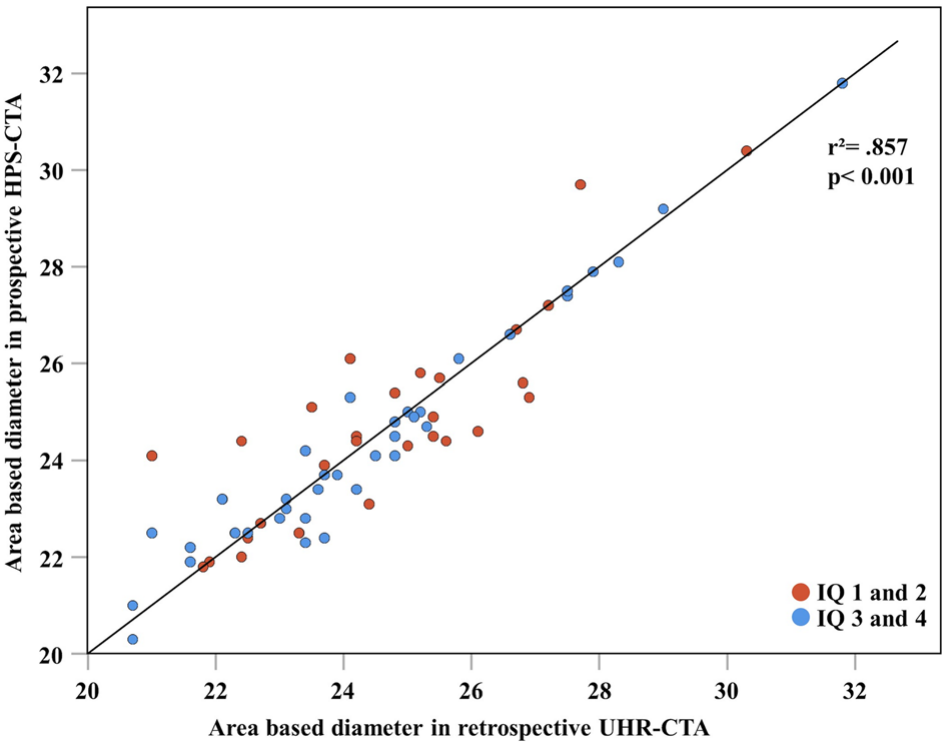
Correlation of area-derived diameter from quantitative measurements obtained from UHR-CTA and HPS-CTA. The overall coefficient of determination was r^2^ = 0.857. For patients with good or excellent image quality, the coefficient of determination was r^2^ = 0.944, and for patients with fair or poor image quality, r^2^ = 0.712

Mean quantitative measurements of AAA were 477.4 ± 91.1 mm^2^ on UHR-CTA and 476.5 ± 90.4 mm^2^ on HPS-CTA, with a mean AAA-difference between both acquisition techniques of 22.3 ± 24.6 mm^2^. A detailed comparison of patient characteristics with AAA differences below and above the mean difference of 22 mm is provided in Supplementary Table S1. The mean AAP for UHR-CTA was 78.3 ± 7.3 mm, whereas HPS-CTA displayed a mean AAP of 78.8 ± 7.3 mm with a mean difference of 1.9 ± 2.2 mm. In multiple linear regression analysis with the magnitude of AAA difference between UHR-CTA and HPS-CTA as the outcome of interest, the aortic annular subjective image quality score was the only predictor with statistical significance (Table 3). AAA-derived diameter measurements obtained from UHR-CTA and HPS-CTA showed strong, positive correlation (Pearson’s r^2^ = 0.86, p < 0.001) (Fig. 3). In patients with good or excellent annular image quality (n = 36), the correlation was r^2^ = 0.94, and in patients with fair or poor image quality (n = 28), the correlation was r^2^ = 0.71 p < 0.001, respectively.

**Table 3:** Multiple linear regression analysis with the difference between AAA measurements as the outcome of interest

Linear regression	Difference in aortic annular area	
	β	p-value
BMI (kg/m^2^)	−0.32	0.1
CTDIvol (mGy)	0.25	0.2
HF mean (bpm)	−0.08	0.5
HF-Variability	−0.005	0.97
Average HU attenuation	0.26	0.09
CNR	−0.07	0.6
Subjective Image Quality of HPS-CTA	0.5	** < 0.001**

The regression model was significant (r^2^ = 0.23, p = 0.03)

*BMI* body-mass-index; *CTDIvol*, Dose CT Volume Index; *HF* heart frequency, *HU* Hounsfield Units; *CNR* contrast-to-noise ratio

HPS-CTA average capture time of the aortic annulus was 209 ± 37 ms. R–R timepoints used for annular assessment on UHR-CTA were on average at 202 ± 48 ms. The mean difference in R–R interval timing between the two methods was 45 ± 39 ms. Differences in R–R interval timing between HPS-CTA and UHR-CTA did not correlate with differences in AAA- and AAP-measurements ((r = −0.09; p = 0.50, and r = −0.09; p = 0.44, respectively).

**Fig. 4:**
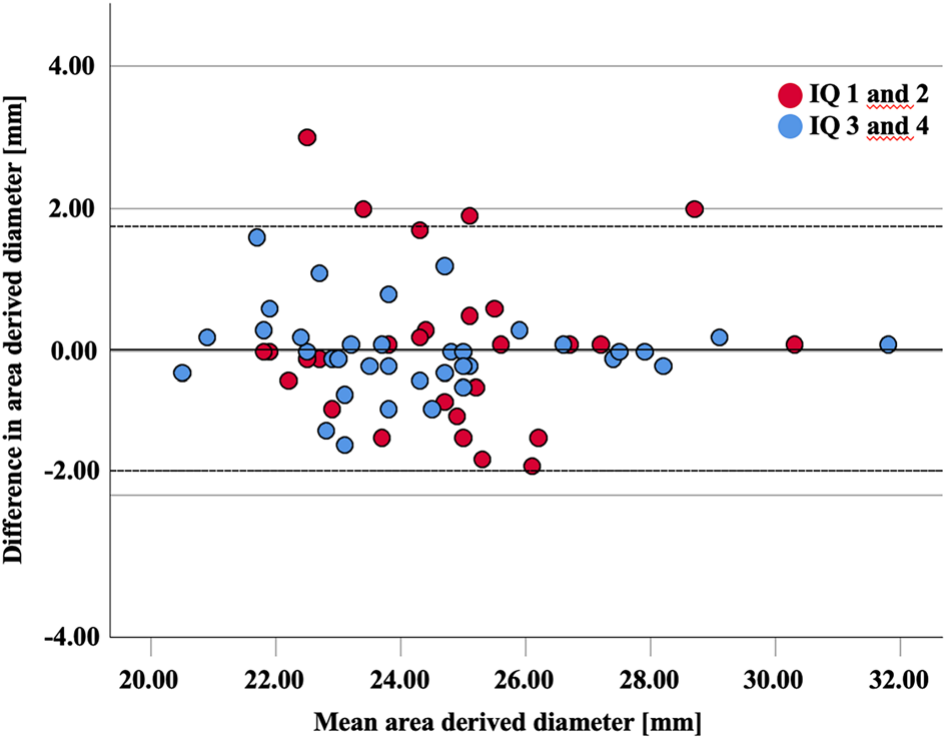
Bland–Altman plot shows measurements of mean area derived diameters performed on high-pitch spectral CT-Angiography (HPS-CTA) compared to Ultrahigh-resolution CT-Angiography, serving as the reference standard. No systematic bias between both methods is observed. The difference between the measurements do not vary systematically with the size of the measurements. A very strong agreement can be appreciated when HPS-CTA presented high subjective image quality (score values 3 and 4–lower and upper limits of −1.2 and 1.1 mm when). For patients with fair or poor image quality (score values of 2 or 1), a larger limit of agreement is noticed (lower limit and upper limit of −2.2 and 2.3 mm, respectively)

After stratification for subjective image quality of aortic annulus depiction on HPS-CTA, Bland-Altmann analysis for patients with good or excellent image quality (n = 36) revealed a mean difference magnitude of 0.40 mm and limits of agreement ranging from −1.2 to 1.1 mm, suggesting negligible bias and small variability. However, for patients with fair or impaired image quality (n = 28), the mean difference magnitude was larger (0.82 mm), with wider limits of agreement: −2.2 to 2.3 mm, indicating increased variability between UHR-CTA and HPS-CTA measurements in lower-quality images. The mean of the absolute diameter differences was close to zero, indicating no systematic smaller or larger values with HPS-CTA compared to UHR-CTA for any image quality group (Fig. 4).

### Hypothetical aortic valve prosthesis selection

The selection concordance for Sapien 3 valves (AAA-based sizing) was 91% (58 out of 64 patients), while for Evolute R valves (AAP-based sizing), it was at 89% (57 out of 64 patients) (Tables 4 and 5). Patients with diverging AAA-based and AAP-based heart valve prosthesis selections showed reduced subjective image quality (median 3 [IQR 2–3] vs. 2 [IQR 1–2], p = 0.048; median 3 [IQR 2–3] vs. 2 [IQR 1–3], p = 0.049). In subjects with overall lower image quality of HPS-CTA (Score value 1 and 2 n = 28) the concordance for both Sapien 3 and Evolute R prosthesis valves were identical at 82% (23 out of 28 patients). Conversely, in patients with high image quality of HPS-CTA (Score value 3 and 4, n = 36), 97% (35/36 patients) had an identical selection for Sapien 3 valves, and 94% (34/36 patients) for Evolute R valves (Table 6). A dedicated analysis of quantitative measurements and subjective image quality of patients with diverging AAA and AAP-based heart valve prosthesis recommendations is given in Supplementary Table S3 and Supplementary Table S4.

**Table 4:** Aortic valve prosthesis selection for SAPIEN 3 valve

		Area based prosthesis sizing for balloon-expandable SAPIEN 3			
		UHR–CTA			
	Valve size	20 mm	23 mm	26 mm	29 mm
*HPS–CTA*	20 mm	1	0	0	0
	23 mm	0	18	2	0
	26 mm	0	2	28	2
	29 mm	0	0	0	11

**Table 5:** Aortic valve prosthesis selection for Evolute R valve

		Perimeter based prosthesis sizing for self-expandable Evolute R			
		UHR–CTA			
	Valve size	23 mm	26 mm	29 mm	34 mm
*HPS–CTA*	23 mm	0	0	0	0
	26 mm	0	12	1	0
	29 mm	0	3	30	1
	34 mm	0	0	2	15

*UHR****–****CTA* Ultrahigh-resolution CT-Angiography; *HPS****–****CTA* High-pitch spiral CT-Angiography

**Table 6:** Identical Prosthesis valve selection in UHR-CTA and HPS-CTA based on HPS-CTA image quality

	Identical prosthesis sizing	
	Sapien 3	Evolute R
Total (*n* = *64*)	58/64 (91%)	57/64 (89%)
High IQ, (*n* = *36*)	35/36 (97%)	34/36 (94%)
Low IQ (*n* = *28*)	23/28 (82%)	23/28 (82%)

Table presents a comparison of identical prosthesis valve selections for Sapien 3 and Evolute R, based on Ultra-High Resolution Computed Tomography Angiography (UHR-CTA) and High-Pitch Spectral CT-angiography (HPS-CTA). The data is categorized into two groups: patients with high image quality score on HPS-CTA (score values 3 and 4) and patients with low image quality on HPS-CTA (score values 1 and 2). The selection criteria for Sapien 3 valves are based on the aortic annular area, whereas for Evolute R, the criteria depend on the aortic valve perimeter

*IQ* Image Quality

## Discussion

In this retrospective study of a prospective cohort, we aimed to evaluate the UHR-CTA and HPS-CTA acquisition techniques on a first-generation dual-source PCD-CT scanner in the context of preprocedural planning for TAVR. Our study hereby focused on image quality and the effect on quantitative measurements of the aortic annulus with a hypothetical prosthesis sizing: Here, we observed that both techniques provide comparable and reliable quantitative aortic annular assessments in most cases, albeit with superior image quality using the UHR-CTA scan mode with guideline-recommended retrospective ECG-gating, but lower radiation dose for prospectively ECG-triggered HPS-CTA.

### Image quality and radiation dose

We observed that UHR-CTA demonstrated superior image quality scores of the aortic annulus compared to HPS-CTA, which aligns with prior studies noting superior image quality and depiction of small details using ultrahigh-resolution photon-counting CTA for coronary arteries [20, 21], for pulmonary imaging [22], and temporal bone imaging [23]. The HPS-CTA features inherent spectral data using novel Quantum Imaging technology and showed reduced image noise compared to UHR-CTA. These results are confirmatory to previous studies focusing on spectral PCD-CT showing image noise reduction [24–26], and to a study recognizing elevated noise levels in ultrahigh-resolution CTA [14]. However, the difference in image noise observed might results from the different nature of the ECG-synchronized acquisition modes: For the ECG-triggered HPS-CTA all the dose is going into a single cardiac phase, while for the retrospective ECG-gated low-pitch UHR-CTA the total dose is distributed over a wider range of cardiac phases, so the images from a certain reconstructed phase only contain a part of the total radiation dose [27]. HPS-CTA using prospective ECG-triggering had a significant advantage in terms of radiation exposure compared to retrospective ECG-gated low-pitch UHR-CTA, confirming well-established research knowledge [28]. Albeit radiation concerns are largely negligible in this elderly patient population, increasing evidence for the beneficial use of TAVR in low-risk individuals [6, 29], which is likely to expand the indication to younger patients [30], who would benefit from low-radiation dose protocols.

### Quantitative measurements of the aortic annular area and the aortic annular perimeter

Regarding the AAA and AAP measurements, we noticed a mean difference of 22.3 ± 24.6 mm^2^ and 1.9 ± 2.2 mm, respectively, with a strong positive correlation (Pearson r^2^ = 0.857). However, we observed a lower correlation regarding quantitative aortic annulus assessments for patients with reduced image quality of HPS-CTA. Surprisingly, we did not find a significant association between image quality and mean heart rate or heart rate variability using the high-pitch CTA, which differs from the results reported by Capilli et al. using a second-generation dual-source CT-scanner [31]. One explanation could be improved temporal resolution and wider detector coverage that came with third generation dual-source CT scanners and that also apply to dual-source photon-counting CTA and better delineation of the annulus, enabling diagnostic image quality over a larger range of heart frequencies [16, 32]. However, intraluminal attenuation was associated with reduced image quality on HPS-CTA. It remains to be seen whether monoenergetic reconstructions with lower keV might have a beneficial influence on image quality and AAA and AAP measurements for TAVR-planning, as contrast signal and luminal attenuation can be increased by employing this technique [33].

### Hypothetical aortic valve prosthesis selection

Despite the small differences in AAA and AAP measurements, we found that the vast majority of patients had identical valve prosthesis sizing with both acquisition techniques (over 89% agreement in AAP- and over 91% in AAA-based sizing), supporting in principle their robustness and suitability for TAVR-planning. However, the aortic annulus undergoes conformational and pulsatile changes between systole and diastole, which can lead to different valve prosthesis size selections when measurements are not performed in the systole [34]. This is particularly important, as over- or under-sizing is associated with complications of TAVR, such as device migration, annulus rupture, paravalvular regurgitation, and hemodynamically relevant leaflet thrombosis [35–37]. Nonetheless, it is crucial to note that patients with different valve prosthesis recommendations based on AAA and AAP measurements had significantly reduced subjective image quality. Therefore, when the image quality of HPS-CTA is suboptimal, clinical decision-making might be affected, emphasizing that the depiction of the aortic annulus in good image quality is paramount in TAVR-planning. Given the potential implications of image quality on valve prosthesis sizing, efforts should be made to improve image quality, especially for HPS-CTA.

### Potential clinical implications

Our study underscores the importance of tailored imaging in TAVR planning, advocating the combined use of UHR-CTA and HPS-CTA—as each offer unique advantages. We suggest a stepwise approach, primarily employing HPS-CTA for the vast majority of cases, with UHR-CTA reserved for instances where enhanced image detail is necessary. UHR-CTA’s superior image quality is invaluable in complex cases for precise valve prosthesis sizing. On the other hand, HPS-CTA’s lower radiation dose is particularly beneficial for younger patients or those requiring multiple scans, aligning with TAVR's expanding indications to lower-risk groups.

### Limitations

Several limitations within our study need acknowledgment: First, the small sample size of 64 patients, predominantly of older age (mean age 81.4 ± 6.9 years), might restrict the broader generalizability of our results. Second, being a single-center study, our findings could be influenced by specific protocols, equipment unique to our center. Third, due to the study's retrospective nature, no randomization in applying UHR-CTA and HPS-CTA was performed. Due to the sequential acquisition of both scans there is a resulting difference in bolus timing and consequently variation in lumen attenuation of the aortic annulus, which might influence the comparability. Lastly, hypothetical prosthesis sizing was based on the measurements of AAA and AAP, real-world decision-making regarding prosthesis sizing involves consideration of other patient-specific factors. In line with updated guidelines, annular measurements from retrospectively ECG-synchronized CTA were used as the reference standard in our comparative analysis, although referring to the ultimately selected prosthesis valve size would have been ideal. Furthermore aortic valve configuration and feasibility of trans-arterial access are relevant for pre-TAVR CT evaluation, but were not part of our analysis.

## Conclusions

To conclude, UHR-CTA and HPS-CTA provide reliable aortic annular assessments for TAVR planning. UHR-CTA offers superior image quality, while HPS-CTA has a lower radiation dose. However, severely impaired image quality on HPS-CTA may influence prosthesis sizing decisions, suggesting that immediate post-scan evaluations could help determine the need for a complementary UHR-CTA.

### Supplementary Information

Below is the link to the electronic supplementary material.

Supplementary file1 (DOC 987 kb)

## Data Availability

Data generated or analyzed during the study are available from the corresponding author by request.

## Abbreviations

UHR-CTA: Ultrahigh-resolution CT angiography
HPS-CTA: High-pitch spectral CT angiography
ECG: Electrocardiogram
PCD-CT: Photon-counting detector CT
TAVR: Trans-catheter aortic valve replacement
AAA: Aortic annulus area
AAP: Aortic annulus perimeter
ROI: Region of interest
HU: Hounsfield Units
CTDIvol: Volume CT Dose index
DLP: Dose Length Product
