# Supplementary material for: Photon-counting CT-angiography in pre-TAVR aortic annulus assessment: effects of retrospective vs. prospective ECG-synchronization on prosthesis valve selection
Source: Int J Cardiovasc Imaging. 2024 Feb 15;40(4):811–20. doi: 10.1007/s10554-024-03050-w (PMC11052843; doi:10.1007/s10554-024-03050-w)
Supplement: Supplementary file 1 — Supplementary file1 (DOC 987 kb) [file 10554_2024_3050_MOESM1_ESM.docx]

**Supplementary Material**

**
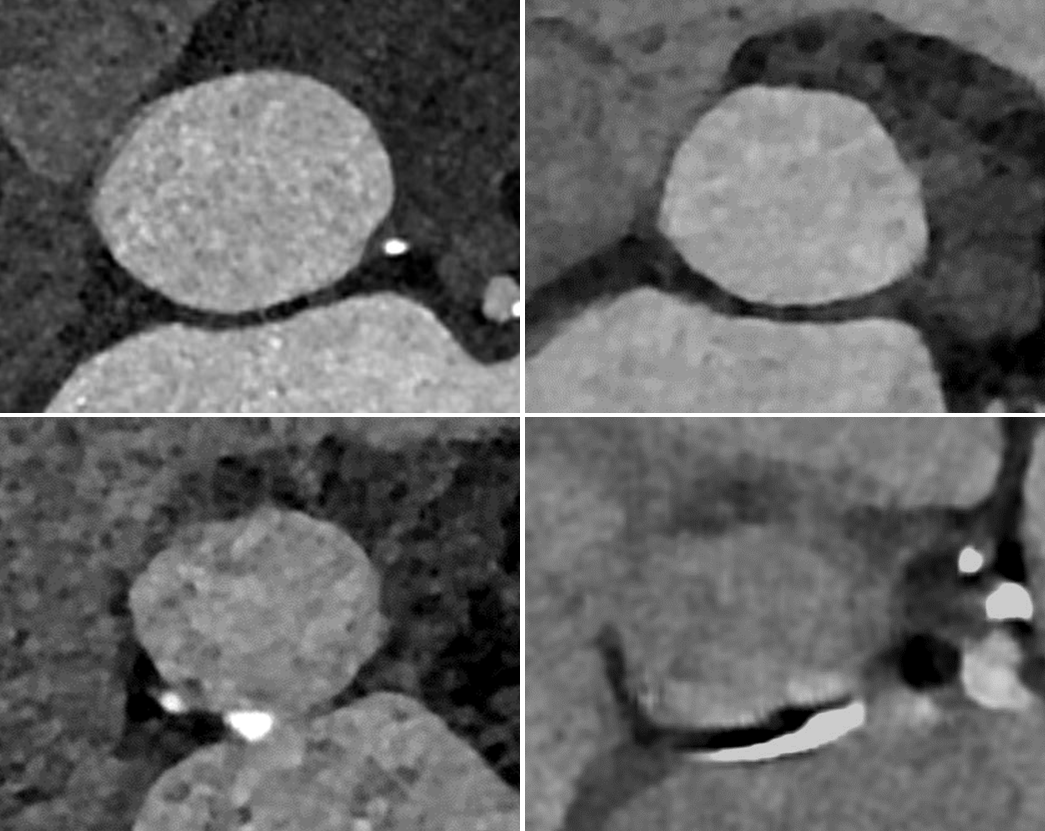
**

**D**

**C**

**B**

**A**

**Figure S1** Comparative examples of subjective image quality grading for aortic annulus HPS-CTA images. A: Excellent (Score of 4) - Clear and artifact-free depiction of the aortic annulus with perfect lumen and margin visibility. B: Good (Score of 3) - Minor artifacts present at the margin, but does not significantly impair the visibility of the aortic annulus. C: Fair (Score of 2) - Detectable artifacts that somewhat interfere with the image, making the lumen or margin less clear. D: Poor (Score of 1) - High presence of motion and blooming artifact interference with blurred depiction of the lumen and margin, significantly impairing the assessment.

**Table S1**

Multiple linear regression analysis. The image quality of high pitch mode for annular measurement as the outcome of interest. The regression model was significant (r²=0.22, p=0.02). Average HU attenuation was the only significant parameter that had an influence on the image quality of high pitch mode (p=0.007).

|  | **Image Quality** |  |
| --- | --- | --- |
| Linear Regression | ***β (95%-CI)*** | ***p-value*** |
| BMI (kg/m²) | 0.25 | 0.2 |
| CTDI_vol (high pitch mode) | 0.09 | 0.6 |
| HF mean | -0.085 | 0.5 |
| HF variability | 0.04 | 0.7 |
| Average HU attenuation  (high pitch mode) | -0.39 | **0.007** |
| CNR (high pitch mode) | 0.19 | 0.2 |

**Table S2**

Comparative analysis of patients with annular area differences below or above the average difference (22 mm). Subjective annular image quality was the only parameter that differed significantly between both groups.

| Difference in AAA | ***≤ 22 mm*** | ***> 22 mm*** | ***p-value*** |
| --- | --- | --- | --- |
| N | 42 | 22 |  |
| BMI (kg/m²) | 26.9 ± 4.3 | 26.8 ± 5.2 | 0.5 |
| CTDI_vol (high pitch mode) | 4.5 ± 1.2 | 4.9 ± 1.3 | 0.14 |
| Heart rate (beats per minute) | 73.6 ± 13.6 | 72.1 ± 11.7 | 0.3 |
| HR variability | 0.24 ± 0.23 | 0.23 ± 0.23 | 0.4 |
| Patients with Arrhythmia | 12 (29%) | 5 (23%) | 0.43 |
| Average HU attenuation | 319 ± 71 | 335 ± 107 | 0.24 |
| CNR | 18.8 ± 6.1 | 19.3 ± 7.2 | 0.4 |
| Image Quality Score | 2 [2;3] | 3 [2;4] | **0.003** |

Data are presented in numbers and frequencies in parenthesis, mean ± standard deviation, and median and interquartile range in square brackets.

Abbreviations: AAA = aortic annular area, BMI = body-mass-index, HR = heart rate, HU = Hounsfield Units, CNR = contrast-to-noise ratio.

**Table S3**

Subgroup analysis: Divergent AAA-based prosthesis valve selection (SAPIEN 3)

| ***Image quality Score*** | ***Area difference (mm²)*** | ***Diameter difference (mm)*** |
| --- | --- | --- |
| 3 | 48 | 1.3 |
| 2 | 46 | 1.2 |
| 2 | 66 | 1.6 |
| 2 | 108 | 3.0 |
| 1 | 51 | 1.3 |
| 1 | 74 | 2.0 |

Table S3 represents a subgroup analysis for divergent AAA-based prosthetic valve selection using the SAPIEN 3 model. The data illustrates the relationship between image quality scores and the observed differences in valve sizing between UHR-CTA and HPS-CTA. The measurements indicate that lower image quality scores were associated with discrepancies in area-based prosthetic valve sizing.

**Table S4**

Subgroup analysis: Divergent AAP- based prosthesis valve selection (Evolute R)

| ***Image quality Score*** | ***Perimeter difference (mm)*** | ***Diameter difference (mm)*** |
| --- | --- | --- |
| 3 | 3.0 | 1.1 |
| 3 | 3.2 | 1.1 |
| 2 | 6.6 | 1.9 |
| 2 | 10.0 | 3.0 |
| 1 | 2.3 | 0.6 |
| 1 | 4.0 | 1.2 |
| 1 | 8.7 | 2.0 |

Table S4 shows a subgroup analysis for divergent AAP-based prosthetic valve selection using the Evolute R model. Divergent perimeter-based hypothetical prosthesis valve sizing was increasingly observed when HPS-CTA showed reduced image quality.
